# Arbuscular Mycorrhizal Fungi Boost Development of an Invasive *Brassicaceae*

**DOI:** 10.1111/pce.15508

**Published:** 2025-03-25

**Authors:** Josh Trombley, John L. Celenza, Serita D. Frey, Mark A. Anthony

**Affiliations:** ^1^ Center for Soil Biogeochemistry and Microbial Ecology, Department of Natural Resources and the Environment University of New Hampshire Durham New Hampshire USA; ^2^ Department of Biology Boston University Boston Massachusetts USA; ^3^ Center for Microbiology & Environmental Systems Science University of Vienna Vienna Austria

**Keywords:** arbuscular mycorrhizal fungi (AMF), garlic mustard (*Alliaria petiolata*), glucosinolates, invasive species management, plant‐fungal interactions

## Abstract

Invasive plant growth is affected by interactions with arbuscular mycorrhizal fungi (AMF). AMF are mutualists of most land plants but suppress the growth of many plants within the *Brassicaceae*, a large plant family including many invasive species. *Alliaria petiolata* (garlic mustard) is a nonnative, nonmycorrhizal *Brassicaceae* distributed throughout North America in forest understories where native species rely on AMF. If AMF suppress growth of garlic mustard, it may be possible to inoculate AMF to manage invasions. Here, we show that in contrast to expectation, garlic mustard growth nearly doubled in response to AMF inoculation under both laboratory and field conditions. This effect was negatively linked to investments in glucosinolates, a class of defensive compounds. In contrast to typical symbiosis, AMF did not produce arbuscules where nutrient exchange occurs in roots, but AMF inoculation increased plant and soil nitrogen availability. Our findings reveal an adjacent pathway by which AMF promote invasive plant growth without classic symbiotic exchanges. Prior assumptions that garlic mustard suppresses AMF are inadequate to explain invasion success since it benefits from interactions with AMF. This study is the first to demonstrate extensive growth promotion following AMF inoculation in mustard plants, with important implications for invasion biology and agriculture.

## Summary statement

- We show that an invasive, “nonmycorrhizal” *Brassicaceae* (*Alliaria petiolata*) can be colonised by AMF, and that this boosts its growth and nutrition even in the absence of classic nutrient exchange structures, arbuscules.

## 1 Introduction

Invasive species threaten the health and biodiversity of terrestrial ecosystems worldwide. Throughout much of the temperate region, most understory (Brundrett and Kendrick 1988) and many dominant overstory tree species form mutualistic associations with arbuscular mycorrhizal fungi (AMF) (Harley and Harley 1987). AMF make significant contributions to plant productivity (Hoeksema et al. 2010), diversity (van der Heijden et al. 2015), and nitrogen (N) and phosphorous (P) cycling (Van Der Heijden et al. 2006). Disturbances to AMF can therefore inhibit native plants highly reliant on this symbiosis (Roberts 1997; Stinson et al. 2006). Members of the mustard family (*Brassicaceae*), which are primarily non‐mycorrhizal and include many invasive species (Meekins et al. 2001; Trautwig et al. 2022; Trautwig et al. 2023), can inhibit AMF, and this is thought to be a key mechanism of invasion success (Stinson et al. 2006; Cipollini and Cipollini 2016). While AMF generally promote native host plant growth, they can colonise *Brassicaceae* roots by forming ‘rudimentary arbuscular mycorrhizae’ where colonisation occurs but does not involve nutrient exchange nor benefit to the plant (Fernández et al. 2019; Mandáková et al. 2019). For some mustard plants, AMF can even have a suppressive effect on plant growth (Veiga et al. 2013; Anthony et al. 2020). Boosting AMF levels in invaded areas may therefore not only promote native plant growth, but it could also suppress invasive mustard plants from further invasion.

To test if supplemental AMF can suppress the growth of invasive mustards, we focused on the nonmycorrhizal, invasive species *Alliaria petiolata* (garlic mustard) (Cipollini and Cipollini 2016). Garlic mustard is allelopathic, disrupts native mycorrhizal associations (Stinson et al. 2006; Anthony et al. 2017), and specifically produces the glucosinolate sinigrin which has been shown to inhibit mycorrhizal spore germination (Cantor et al. 2011). Garlic mustard itself has long been assumed to be non‐mycorrhizal (Poon and Maherali 2015), like most members of the *Brassicaceae* (Delaux et al. 2014; Brundrett and Tedersoo 2018). However, in a previous study by Anthony et al. (2020), AMF inoculation nearly halted reproduction in the *Brassicaceae Arabidopsis thaliana* (a phylogenetically related species to garlic mustard). Negative effects of AMF were only observed on mutant *Arabidopsis* lines that no longer produce indolic glucosinolates. This raises the possibility that direct AMF inoculation could be used to suppress growth and reproduction in invasive garlic mustard if glucosinolate levels are low enough, thereby facilitating restoration using microbiome manipulation.

**Figure 1:**
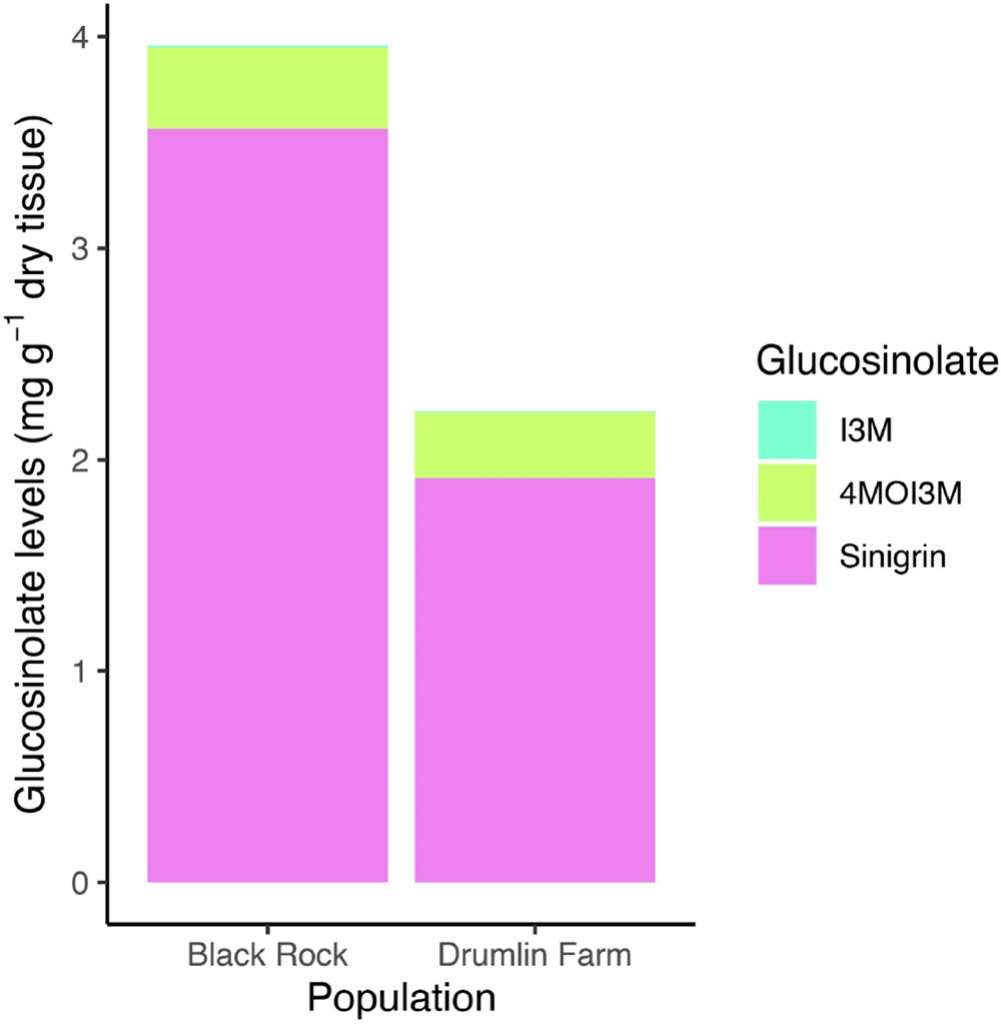
Glucosinolate profiles in the two garlic mustard populations. The three most concentrated glucosinolates are shown, including 4‐methoxy‐indol‐3‐ylmethyl glucosinolate. (4MOI3M), indol‐3‐ylmethyl glucosinolate (I3M), and 2‐propenyl‐glucosinolate (sinigrin). Sinigrin is found at higher levels than I3M and 4MOI3M in both populations. Bars show the mean of eight replicate values in control treatments without AMF inoculation.

To directly measure the effects of AMF on garlic mustard growth and development, we first conducted a laboratory experiment in which we inoculated garlic mustard plants sourced from two geographically distinct populations with multiple levels of AMF inoculant. The two populations are separated by 365 km; the invasion at Black Rock is denser (Anthony et al. 2017) and at least two times older than at Drumlin Farm. Importantly, glucosinolate levels are nearly 50% higher in the Black Rock compared to Drumlin Farm population, offering a unique chance to test how glucosinolate levels mediate response to AMF inoculation (Figure 1). After 80 days of growth, we measured root and shoot biomass, glucosinolate production, AMF root colonisation, plant tissue nutrients, reproductive output, and rhizosphere soil characteristics. Because AMF can impede growth and reproduction in close relatives of garlic mustard (Veiga et al. 2013; Fernández et al. 2019; Anthony et al. 2020), we hypothesised that AMF inoculation would also inhibit growth and development of garlic mustard relative to control treatments without AMF inoculation.

## 2 Materials and Methods

### 2.1 Laboratory Study

Experimental design: To examine the interactive effects of AMF inoculation and population on garlic mustard growth, AMF colonisation, and glucosinolate production, we grew garlic mustard sourced from two geographically distinct populations in laboratory conditions inoculated with one of three levels of AMF. To ensure the establishment of mycorrhizae adjacent to garlic mustard plants, we grew garlic mustard alongside a common AMF‐associated plant (*Daucus carota*, carrot) (*sensu* Veiga et al. 2013). Garlic mustard was grown in split pots separated using nylon mesh sheets (50 µM) to ensure that carrot and garlic mustard roots did not come into direct contact, but AMF hyphae could freely grow into both compartments (see below). After the garlic mustard plants reached sexual reproduction and began to produce siliques with visible seeds, we destructively harvested all garlic mustard and carrot plants.

We measured plant growth, AMF colonisation, glucosinolate production, and tissue nutrients on the garlic mustard individuals. The experiment included two categorical treatment variables each with ten replicates: (1) an AMF treatment with three levels: control (no AMF), low (100 spores cm^−^^3^ soil), and high (200 spores cm^−^^3^ soil); and (2) population with two levels: Black Rock and Drumlin Farm. Plant‐growth response variables included below‐ and aboveground (i.e., root and shoot) biomass for both garlic mustard and carrot; AMF colonisation response variables included percent hyphae, vesicles, arbuscules, and total AMF root colonisation for both plants; glucosinolate response variables included total production of sinigrin in garlic mustard roots and shoots, and glucobrassicin (indol‐3‐ylmethyl glucosinolate; I3M) and 4‐methoxyglucobrassicin (4‐methoxyindol‐3‐ylmethyl glucosinolate; 4MOI3M) in roots only; tissue nutrient response variables included absolute and percent nitrogen and phosphorous content.

Garlic mustard seeds used in the laboratory experiment were collected from dozens of plants at populations from two invaded forest sites in the northeastern U.S.A. The Black Rock Forest in Cornwall, New York has been invaded by garlic mustard for more than 65 years (Lankau 2011), and the Drumlin Farm Wildlife Sanctuary in Lincoln, Massachusetts has been invaded for roughly 35 years (discussion with land managers). We collected siliques (the fruiting bodies of garlic mustard) from plants at each site between seed maturation and the first snowfall (i.e., August–November), separated the seeds from the dried valves, and stored them at 4°C. Tendersweet Carrot seeds were obtained from Sammy's Seeds LLC (Hugo, Minnesota, USA).

We obtained *Rhizophagus irregularis* (culture‐line DAOM197198, DAOM181602, and MUCL43194) from Symplanta (Graupnerweg, Germany). The inoculum contained 1 million spores in 250 g of food‐grade diatomaceous earth. Growing medium was composed of 50% Miracle‐Gro Premium Potting Mix (Marysville, Ohio, USA) and 50% QUIKRETE all‐purpose sand (Atlanta, Georgia, USA) by volume. We sterilised the growing medium by autoclaving consecutively three times for 45 min at 121°C. Autoclaving has effects that alter the soil matrix, including modifying organic matter composition and releasing nutrients by lysed microbial cells. While this does not affect our ability to compare control and inoculated conditions, it may create artificial growth conditions that alter the growth of both garlic mustard and *Rhizophagus irregularis*.

Garlic mustard seeds require cold stratification to germinate. We stored seeds in humid conditions at 4°C for 28 days, scarified the seeds by lightly scraping the seed coat, and incubated in 2 mM gibberellic acid for 48 h at 15°C on moist paper towels. We have previously grown garlic mustard from seed to verify that this approach promotes germination. After germination, seeds were transferred to sterile growth medium in flat seedling trays for further development. We then direct seeded carrots into separate seedling trays.

Once the first true leaves had formed on > 50% of plants, we transferred them to the final growth pots. We grew one individual of each species in 4‐inch square plastic pots containing 250 cm^3^ of growth medium at 25°C with 50% humidity on a 12 h on:off light cycle each day. Each pot was bisected by a 50 µm nylon mesh to separate the root systems of the two plants while allowing free infiltration of fungal hyphae (Bingham and Simard 2011). The point of this design was to grow a plant that supported AMF growth in the microcosms without direct root‐root competition/facilitation to avoid additional levels of complexity. The goal was not to test for common mycorrhizal networks though they may have formed in the experiment. We inoculated with AMF at two levels, both exceeding those found in field soils (Yang et al. 2011; Aleixo et al. 2014) for a total of three AMF spore levels: 0 (Control), 100 (Low), and 200 (High) spores per cubic centimetre of growth medium. As the inoculum contained diatomaceous earth, we added sterilised diatomaceous earth to the control and low treatments so that the total amount of diatomaceous earth added to each pot was equivalent.

While AMF and diatomaceous earth may directly add nutrients to the soil, this is not a significant factor. Diatomaceous earth is > 95% silica, aluminium, and iron oxides, and it does not add nitrogen nor phosphorus to the soil. It was also added at equal proportions across control and treatment plots. While we did not inoculate sterilised AMF spores into the control treatment, direct AMF nutrient additions in the treatment plots are negligible in our study. On average, fungal biomass contains 0.6% P and 3.9% N (Zhang and Elser 2017) with a spore mass of 33–200 pg (Sesartic and Dallafior 2011). We added 25 000–50 000 spores to each pot. Assuming an average spore mass of 200 pg because AMF produce relatively large spores, we added approximately 0.2–0.4 µg of N and 0.03–0.06 µg of P to each pot, a negligible addition. We must also consider that inoculated AMF did not immediately turnover following inoculation because we observed high AMF root colonisation on carrot, so fungal‐necromass derived nutrients were also not directly available to garlic mustard.

Harvest, sample processing, and biomass: We harvested the Drumlin Farm and Black Rock populations when vegetative growth had slowed significantly, 81 and 78 days post‐germination, respectively. After taking small samples for downstream analysis, we dried root and shoot tissue at 60°C for 96 h to calculate moisture content and total plant biomass.

AMF colonisation, glucosinolates, and tissue nutrients: We quantified glucosinolate content as described in (Brown et al. 2003). Glucosinolates were isolated using anion exchange (DEAE Sephadex A25). Reverse phase HPLC was carried out using a Waters 2795 HPLC equipped with a Waters 2996 photodiode array detector and a Phenomenex Luna 5 μm 250 × 4.6 mm C18 column. Individual desulfoglucosinolates were confirmed by comparing the retention time and UV spectra to purified standards and quantified at 229 nm relative to an external sinigrin standard. We quantified AMF colonisation using the grid‐intersection method (McGonigle et al. 1990). Roots were cleared at 95°C in 10% KOH solution for 10 min and stained by boiling in a solution of 1% Schaeffer black pen ink (Bangalore, Karnataka, India) in 0.25% acetic acid for 3 min. Stained roots were measured for hyphal, arbuscule, vesicle, and total AMF colonisation after 50 passes at 100× magnification on a compound microscope. As expected, we observed significant mycorrhizal colonisation of host plant (carrot) roots treated with AMF, confirming that AMF inoculation resulted in the development of successful mycorrhizae. In the control (no AMF added), we found no mycorrhizal colonisation in neither garlic mustard nor carrot roots.

We measured total tissue carbon and nitrogen by dry combustion using a Perkins Elmer Elemental Analyser. Tissue phosphorous was measured by acid digestion at the Pennsylvania State University Agricultural Analytical Services Laboratory (University Park, PA, USA).

### 2.2 Field Study

This experiment was conducted at the Lubberland Creek Preserve in Newmarket, New Hampshire, USA. Garlic mustard has been present at this site for 12 years (discussions with land manager). All plots were in a heavily invaded area within 25 m of the preserve entrance.

To examine the effects of AMF inoculation on in situ garlic mustard development, we inoculated invaded plots with AMF and measured the effect of inoculation on garlic mustard growth, reproduction, and AMF root colonisation. We examined the effect of one treatment variable, AMF inoculation with two levels, ‘AMF added’ (1 million spores m^−^^2^) and ‘control’ (0 spores m^−^^2^), on the following response variables: root and shoot biomass for first and second‐year plants, the total number of first‐year plants, silique production (i.e., reproductive output) in second‐year plants, soil ammonium concentration, and tissue glucosinolate, carbon, nitrogen, and phosphorous content.

We established eight 0.25 m^2^ plots with similar garlic mustard density for use in this study. In July 2022, using a randomised design, we inoculated four of each plot with either 250 000 *R. irregularis* spores) suspended in diatomaceous earth (AMF treatment) or pure diatomaceous earth (Control). AMF in diatomaceous earth and control diatomaceous earth were added as an aqueous solution into three 10 cm deep slits to inoculate soil in the rooting zone and not just on the surface soil. Consistent with the laboratory experiment, direct nutrient addition via AMF biomass was negligible in the plots. We added an estimated 8 µg of N m^2^ and 1.2 µg of P m^2^ to each plot.

We harvested all plots in June 2023 after siliques had matured but before seed dispersal. We first removed and counted all non‐garlic‐mustard plants and collected rhizosphere soil. We defined rhizosphere soil as that which is adhered to plant roots after shaking off bulk soil (Micallef et al. 2009). We separated the roots and shoots for all plants and subsampled five first and second year plants at random from each plot. We removed and stored small portions of each sampled plant for downstream analysis, calculated dry biomass, and counted the number of siliques. Methods for quantifying glucosinolates, tissue nutrients, and soil elemental concentrations were identical to those used in the laboratory study. We quantified soil inorganic nitrogen using the procedure outlined elsewhere (Contosta et al. 2011).

### 2.3 Statistical Analyses

All statistical analyses were performed in R version 4.3.3 (R Team 2013). To quantify the effects of the AMF treatment and population on our response variables, we performed analysis of variance using type III sums of squares using the ANOVA function from the car package (Fox et al. 2012). Kruskal‐Wallis rank sum tests were used for the field experiment because there was only one factor with two levels to compare. To examine the relationships between each of our response variables, we performed correlation tests using the corr.test function in the ‘psych’ package with method = ‘pearson’ (Revelle and Revelle 2015). Correlations with AMF colonisation did not include control plants because there was no observed AMF colonisation, and population‐level correlations are displayed separately when correlations were significant in one population but not the other.

To assess whether magnitude of change in plant growth and glucosinolate production following AMF inoculation were linked, we computed response ratios (*sensu* Moore et al. 2021). Response ratios for glucosinolates represent induced production. Response ratios were calculated for the low and high AMF treatments in relation to the median control value respective to each population to control for population level differences in background glucosinolate production.

$$\text{Response}\;\text{ratio}\;=\;\log(\text{Treatment}\;{\text{value}}_{\text{Replicate}\;X}{/\text{Control}}_{\text{Median}\;\text{of}\;\text{uninoculated}\;\text{treatment}\;\text{by}\;\text{population}})$$

We then predicted the plant growth response ratio as a function of induced glucosinolate production using linear regression and the base lm function in R.

## 3 Results and Discussion

### 3.1 AMF Inoculation Boosted Garlic Mustard Growth

**Figure 2:**
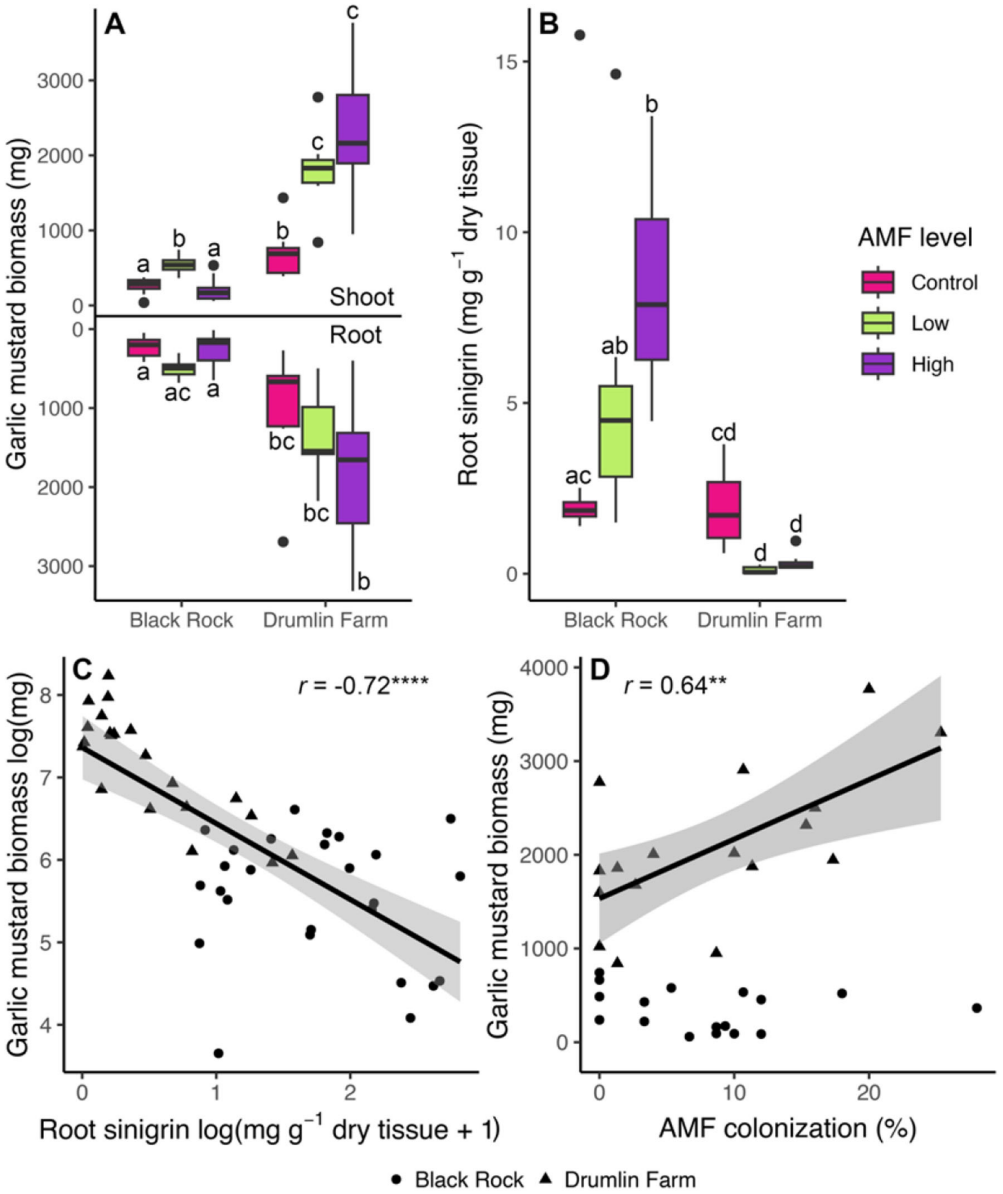
The effect of AMF inoculation and population on garlic mustard biomass and root sinigrin production. Differences in root and shoot biomass across AMF addition levels and between populations (A). Root sinigrin levels across AMF addition levels and between populations (B). Boxes show the interquartile range, horizontal black lines show the mean, upper and lower whiskers show the minimum and maximum values, respectively, and points show outliers. Different lowercase letters indicate significant differences based on Tukey contrasts (*p* < 0.05). See Supporting Information S1: Table S1 for full statistical results. The correlation between garlic mustard shoot biomass and root sinigrin levels (both control and AMF addition treatments (C) and AMF colonisation (only treatments with AMF additions because AMF colonisation was not observed in control plots and only at the Drumlin Farm site because this correlation was not significant for Black Rock (D). Lines show the linear correlation, shaded areas show the 95% confidence interval, and *r* values show the Pearson correlation where ** indicates *p* < 0.001 and **** *p* < 0.00001. [Color figure can be viewed at wileyonlinelibrary.com]

Contrary to expectation, AMF treatment significantly increased overall garlic mustard biomass in both studied populations (Figure 2A, Supporting Information S1: Table S1; see photo in Supporting Information S1: Figure S1). AMF treatments nearly doubled shoot biomass in the Drumlin Farm population, with no effect on root biomass. In the Black Rock population, the low AMF treatment doubled shoot biomass, while the high AMF treatment did not differ from the control (Figure 2A). While the magnitude of these responses differed between the two populations and AMF inoculation levels, these results reveal that garlic mustard generally benefits from AMF inoculation.

**Figure 3:**
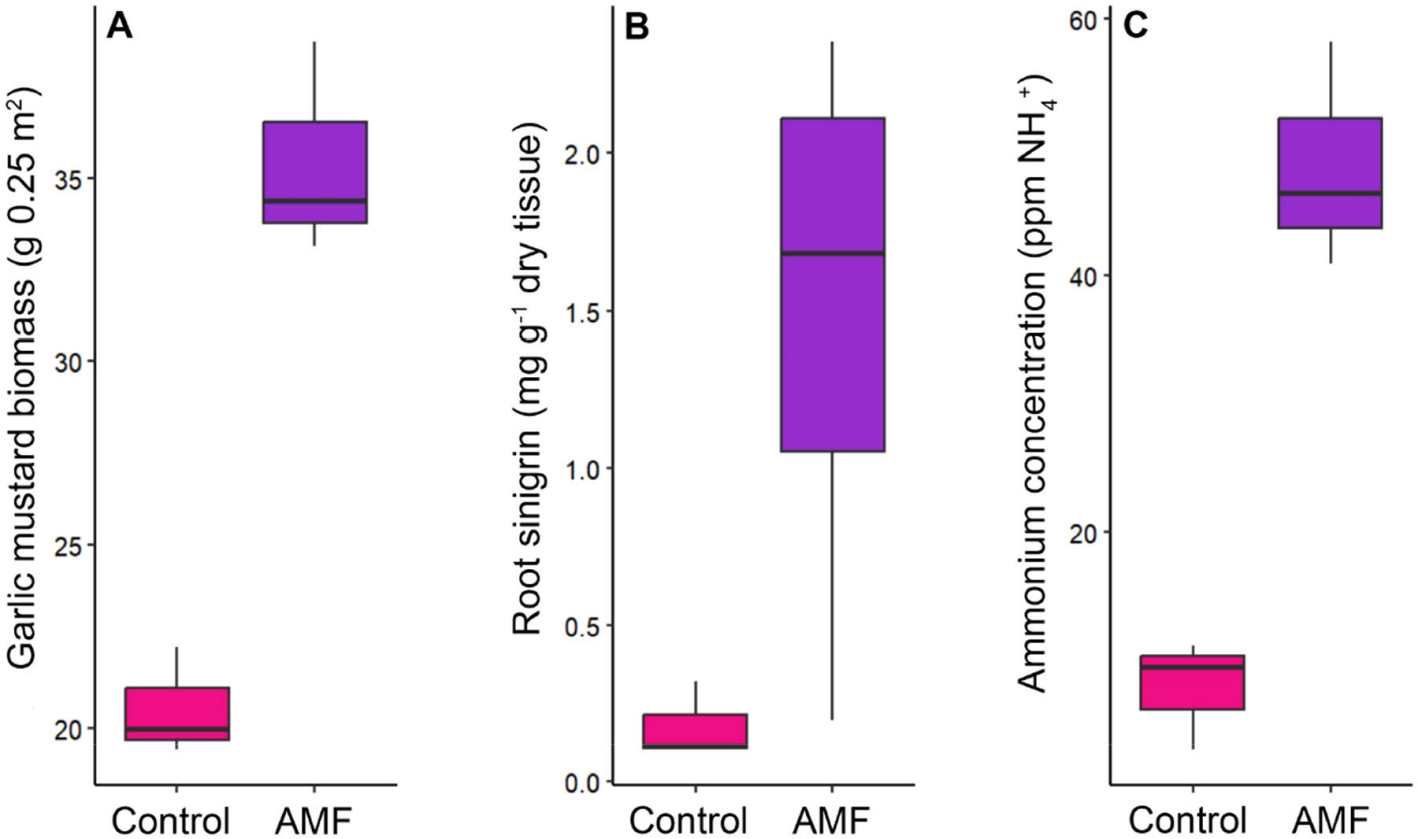
The effects of AMF inoculation in the field study on garlic‐mustard biomass (A), root sinigrin production (B), and in situ rhizosphere soil ammonium concentration (C). AMF treatment significantly increased total biomass (H(1) = 3.9, *p* = 0.05), root sinigrin production (H(1) = 11.86, *p* = 0.001), and rhizosphere soil ammonium concentration (H(1) = 3.9, *p* = 0.05) in garlic mustard. [Color figure can be viewed at wileyonlinelibrary.com]

To examine whether observed effects in the laboratory experiment occur under field conditions where there are already established soil microbiomes, we performed an AMF inoculation experiment at a long‐term garlic mustard invaded forest with a third, distinct geographic population of plants (see Methods and Materials). This location was selected to test how generalisable our findings from the lab may be in a third, independent population where garlic mustard is dense and actively controlled, making this site especially relevant with respect to whether AMF inoculation could be a useful restoration tool. Consistent with our laboratory results, AMF inoculation increased total garlic mustard biomass in reproductive garlic mustard plants by nearly 40% (Figure 3A). Garlic mustard is a biannual in North America (Meekins et al. 2001) and reproducing second‐year plants comprised the majority (> 99% of individuals were second‐year plants) of the biomass in the plots. AMF inoculation therefore increased overall garlic mustard biomass at the plot level.

### 3.2 Glucosinolate Concentrations Influence Growth Responses to AMF Inoculation

Garlic mustard produces glucosinolates, a class of antimicrobial compounds known to disrupt mycorrhizal symbioses (Wolfe et al. 2008; Cantor et al. 2011). To examine the glucosinolate response in garlic mustard plants treated with AMF, we quantified tissue concentrations of three glucosinolates that are the most abundant in garlic mustard: sinigrin, glucobrassicin (I3M), and 4‐methoxyglucobrassicin (4MOI3M) using reverse phase high pressure liquid chromatography (HPLC). Root sinigrin is the most abundant (Figure 1) and best‐studied of these compounds because it inhibits AMF spore germination (Cantor et al. 2011) and disrupts native plant‐mycorrhizal associations (Roberts 1997; Stinson et al. 2006). Production of these compounds in garlic mustard may, in part, be a response to opportunistic colonisation by native mycorrhizal fungi; we thus anticipated that AMF inoculation would stimulate glucosinolate production. We found that AMF inoculation of garlic mustard had a significant effect on root sinigrin, but this effect varied among populations (Supporting Information S1: Table S1). AMF treatment tended to reduce root sinigrin production in the Drumlin Farm population where we observed the largest plant growth promotion in response to AMF, but increased root sinigrin production in the Black Rock population and in the field study (Figure 2B, Figure 3B). Sinigrin levels were significantly increased under the high AMF addition level for the Black Rock population, and this is the only experimental level where garlic mustard biomass was not higher than the control. It is notable that the indolic glucosinolates I3M and 4MOI3M were at much lower concentrations than sinigrin (Figure 1) but followed similar patterns to sinigrin in response to AMF inoculation (Supporting Information S1: Figure S2). Thus, where root sinigrin production remained unchanged or decreased following AMF inoculation, we also observed the greatest increase in plant growth in response to AMF.

**Figure 4:**
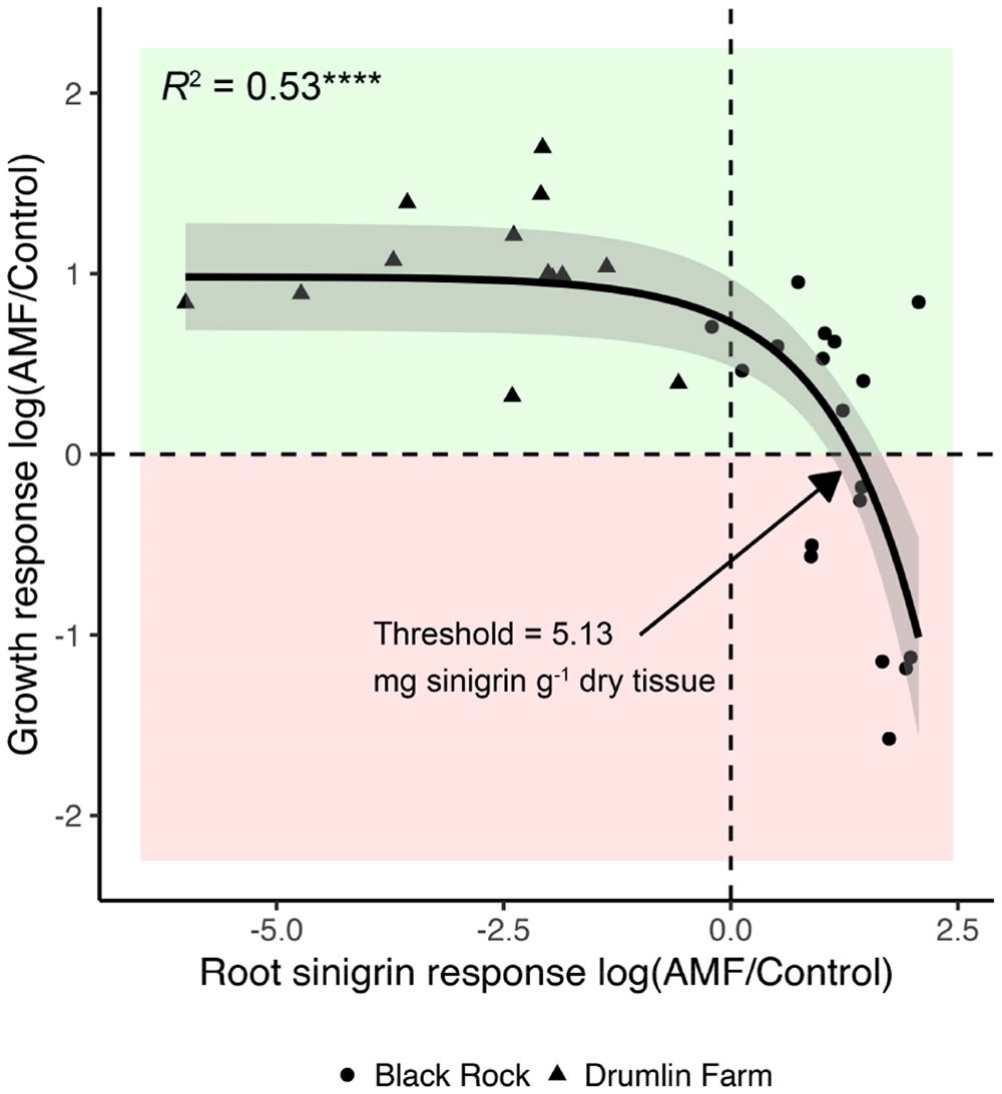
Plant growth responses to AMF inoculation in relation to induced sinigrin production. Each point represents an individual plant in the low and high AMF inoculation treatments. Response ratios were calculated as the log of each replicate value in the AMF inoculation treatments divided by the median control value respective to each population (see methods). Values greater or lower than zero indicate positive and negative growth responses (*y* axis) and reduced or induced sinigrin production (*x* axis) by AMF, respectively. A negative exponential line best captured the relationship, the shaded area shows the 95% confidence interval, and **** = *p* < 0.00001. The predicted threshold of induced sinigrin production where plant growth responses to AMF switched from negative to positive was identified where the exponential line passed zero on the *y* axis. [Color figure can be viewed at wileyonlinelibrary.com]

Production of defence compounds often requires a tradeoff with growth. This ‘growth‐defence trade‐off’ is one of the foundational principles of plant biology (Figueroa‐Macías et al. 2021; He et al. 2022; Malhotra et al. 2023). We therefore expected high levels of glucosinolate production to reduce garlic mustard growth as they are involved in plant defence, including against pathogens (Vega‐Álvarez et al. 2023). In the laboratory study, we found a significant negative correlation between root sinigrin and garlic mustard biomass (Figure 2C). Specifically, plant growth switched from a positive to negative response to AMF when they produced > 5.13 mg sinigrin g^−^^1^ dry tissue (Figure 4). When considered together, plants that produce high levels of sinigrin had a reduced positive plant growth response to AMF inoculation (e.g., the high AMF level for the Black Rock population) likely due to energy constraints of investments in defence, though other mechanisms may further explain this result.

### 3.3 AMF Colonise Garlic Mustard Roots but Do Not Produce Arbuscules

Garlic mustard is assumed to be non‐mycorrhizal (Poon and Maherali 2015), but it grew more in response to AMF inoculation. To examine whether these effects may be a direct outcome of AMF nutrient exchange, we quantified mycorrhizal fungal structures in roots using microscopy (see Methods and Materials). To identify overall colonisation as well as the development of nutrient‐exchange organs (arbuscules), we quantified AMF arbuscules, vesicles, and hyphae. In the laboratory study, AMF inoculation resulted in very low levels of mycorrhizal colonisation (8% ± 1%) compared to the co‐occurring mycorrhizal host plant (54% ± 5%). We observed hyphal and vesicle colonisation localised in isolated patches in garlic mustard root systems. There were no observed instances of arbuscule formation in garlic mustard roots, while arbuscule formation in the mycorrhizal host plant was high (43% ± 5%). In the Drumlin Farm population, where plants were the largest and root sinigrin levels were relatively low, AMF colonisation was positively correlated with garlic mustard growth (Figure 2D). In the Black Rock population, where plants were generally smaller and root sinigrin levels were higher, this relationship was not present. This suggests that higher production of sinigrin disrupts an otherwise positive relationship between AMF colonisation and garlic mustard growth. To that end, in the Black Rock population (where sinigrin levels were high), there was a significant negative correlation between biomass and root sinigrin in the AMF‐treated plants (*r* = −0.54, *p* = 0.02). AMF can therefore colonise garlic mustard roots, and this benefits plants from populations that do not produce high levels of sinigrin even in the absence of nutrient exchange structures (arbuscules).

### 3.4 Tissue Nutrients and Soil Characteristics Can Explain AMF Colonisation

AMF enhance growth in their symbionts by facilitating uptake of key nutrients including nitrogen (Jin et al. 2005) and phosphorous (Javot et al. 2007). In a typical arbuscular mycorrhizal symbiosis, we expect increased phosphorous and nitrogen uptake due to AMF inoculation. To investigate the effects of the AMF treatments on garlic mustard tissue nutrients, we quantified root and shoot nitrogen and phosphorous levels. Despite the absence of arbuscules on garlic mustard root systems, total tissue nitrogen increased with AMF inoculation in both the laboratory and field studies (Supporting Information S1: Figure S3). In the Drumlin Farm population where AMF colonisation was positively correlated with plant growth, both AMF treatment levels significantly increased total shoot nitrogen. In the Black Rock population, only the low treatment increased total root and shoot nitrogen, consistent with observed plant growth responses. In the field, AMF treatment increased total root nitrogen in second‐year plants where growth was also stimulated by AMF inoculation. Across treatments and populations, relative tissue nitrogen (e.g., concentration) remained stable. In other words, as plants grew larger with AMF treatment, they also took up more nitrogen and their stoichiometry was maintained (Supporting Information S1: Figure S4). Contrary to typical mycorrhizal functioning in the field study, AMF treatment reduced relative tissue phosphorous while total tissue phosphorous remained unchanged (Supporting Information S1: Figure S5), consistent with recent observations that AMF likely do not provide phosphorous to nonhost *Brassicaceae* (Wang et al. 2023). As the plants grew larger, they did not take up more phosphorus, which shows that AMF inoculation enhanced garlic mustard nitrogen but not phosphorus uptake.

In the field study, AMF inoculation increased rhizosphere soil ammonium concentration (Figure 3C) and total tissue nitrogen in second‐year plants (Figure 3B). These results demonstrate the AMF inoculation increases available soil nitrogen in the field. This may explain why nitrogen uptake and growth in garlic mustard was enhanced by AMF inoculation. The positive effect of increased soil ammonium on plant growth is well established (Joseph et al. 1976; Schenk and Wehrmann 1979; Lobit et al. 2007). AMF can promote proteolytic enzyme production (de Souza et al. 2007), soil nitrogen mineralisation (Saia et al. 2014) and organic matter decomposition (Hodge et al. 2001) by priming the soil microbial community (Frey 2019). However, the mechanism by which AMF inoculation increased soil ammonium and tissue nitrogen in our study remains unclear. In typical arbuscular mycorrhizal symbioses, nutrient exchange occurs in heavily colonised roots containing arbuscules. We observed very low levels of AMF colonisation in garlic mustard roots compared to typical AMF‐associated plants, and no arbuscules were found. Thus, a typical mycorrhiza is unlikely in this case. We therefore hypothesise that garlic mustard benefits from the presence of AMF through an alternative process that is mechanistically distinct from other forms of mycorrhizae.

### 3.5 *Brassicaceae* Growth Benefits From AMF via an Adjacent Mechanism

It has long been assumed that mycorrhizal mutualisms can inhibit growth in non‐mycorrhizal neighbours (Ocampo 1986; Francis and Read 1994; Rinaudo et al. 2010; Wang et al. 2023). In contrast, we show that AMF inoculation and subsequent root colonisation significantly enhanced growth in garlic mustard. An earlier study demonstrated that AMF inoculation may inhibit garlic mustard when grown without a co‐occurring mycorrhizal neighbour plant (Roberts 1997). In our study, we grew garlic mustard alongside a mycorrhizal plant to support an AMF network because it invades forests with mostly AMF associated co‐occurring plants (Stinson et al. 2007). Neither garlic mustard growth nor glucosinolate production were correlated with AMF colonisation in the adjacent plants in our study (Figure S6), indicating no harm to AMF colonisation. Taken together, these findings suggest a mutually beneficial or at least commensal interaction between garlic mustard and AMF.

Arbuscular mycorrhizal plants may act as nurse plants to enhance garlic mustard growth. Facilitative effects of one plant on the growth of co‐occurring species occurs via a variety of mechanisms (Filazzola and Lortie 2014), including enhanced mycorrhizal fungal colonisation of benefactor plants (Carrillo‐Garcia et al. 1999). However, garlic mustard did not form typical mycorrhizae in our study, with no arbuscule formation and overall low root colonisation levels. An absence of arbuscules in garlic mustard roots distinguishes it from most arbuscular mycorrhizal plants (Brundrett and Tedersoo 2019; Liu et al. 2020). One possibility is that AMF directly provided nutrients to garlic mustard even in the absence of arbuscules (Bueno et al. 2019). *Scutellospora calospora* can transfer phosphorus to reduced mycorrhizal mutants of tomato even without penetrating cortical cells or forming arbuscules (Manjarrez et al. 2010). Another explanation may be that AMF act more like endophytes in garlic mustard roots (Brundrett 2006), stimulating hormone production (Ludwig‐Müller et al. 1997; Sun et al. 2020) and improving nutrient uptake (Newsham 2011). Other *Brassicaceae* produce signalling molecules (strigolactones) which induce hyphal growth in AMF and the formation of mycorrhizal symbioses (Akiyama et al. 2005; Brewer et al. 2012; Wang et al. 2020). Garlic mustard may produce these compounds and induce hyphal growth from adjacent mycorrhizal symbionts towards its own root systems, making nitrogen more available (Hodge et al. 2001). We conceptualise this as an ‘adjacent mycorrhizal symbiosis’ because the non‐mycorrhizal plant still interacts with AMF but benefits distinctly from arbuscular mycorrhizal plants.

In conclusion, our work challenges traditional assumptions about the relationship between garlic mustard, AMF, and native plant communities in its nonnative range. Our findings suggest that garlic mustard benefits significantly from AMF inoculation through a previously unknown mechanism distinct from typical mycorrhizal symbioses, here called ‘adjacent mycorrhizal symbiosis’. The discovery of this class of symbiosis could have far‐reaching implications. The formation of arbuscules is the main criterion used to determine whether a plant is capable of AMF symbiosis (Brundrett and Tedersoo 2019) but see (Bueno et al. 2019). Here, we show that a common invasive plant benefits from AMF without arbuscule development and with low levels of colonisation. Our results have far reaching consequences beyond garlic mustard because major agricultural plants are also members of the *Brassicaceae* (e.g., canola, black mustard, broccoli), and many can be colonised by AMF without arbuscule formation (Demars and Boerner 1996), suggesting they may experience similar feedback. Our findings highlight the need for further research to understand the mechanisms driving these interactions and their implications for invasive species management, forest ecosystem health, and fungal ecology.

## Conflicts of Interest

The authors declare no conflicts of interest.

## Supporting information

Supplementary Materials.

## Data Availability

All data and scripts associated with this manuscript are available in the following GitHub repository: https://github.com/Josh-Trombley/Garlic-Mustard.
