# Supplementary material for: Arbuscular Mycorrhizal Fungi Boost Development of an Invasive Brassicaceae
Source: Plant Cell Environ. 2025 Mar 25;48(7):4928–37. doi: 10.1111/pce.15508 (PMC12131958; doi:10.1111/pce.15508)
Supplement: Supplementary file 1 — Supplementary Materials. [file PCE-48-4928-s001.docx]

**Supplementary Materials**

**Table S1.** Analysis of variance results for garlic mustard biomass and root sinigrin levels in the lab experiment.

| **Shoot biomass** | *Sum Sq* | *Df* | *F-value* | *P-value* |
| --- | --- | --- | --- | --- |
| Intercept | 231.976 | 1 | 811.028 | < 2.2e-16 |
| AMF | 6.48 | 2 | 11.328 | 1.00E-04 |
| Population | 4.76 | 1 | 16.641 | 0.0001776 |
| AMF x Population | 6.254 | 2 | 10.933 | 0.0001305 |
| Residuals | 13.157 | 46 |  |  |
|  |  |  |  |  |
| **Root biomass** |  |  |  |  |
| Intercept | 213.762 | 1 | 387.3234 | <2.20E-16 |
| AMF | 6.656 | 2 | 6.0298 | 0.0047849 |
| Population | 9.981 | 1 | 18.0846 | 0.0001053 |
| AMF x Population | 4.701 | 2 | 4.259 | 0.0202326 |
| Residuals | 24.835 | 45 |  |  |
|  |  |  |  |  |
| **Root sinigrin** |  |  |  |  |
| Intercept | 101.68 | 1 | 10.6856 | 0.00219 |
| AMF | 113.28 | 2 | 5.9519 | 0.005378 |
| Population | 10.89 | 1 | 1.1444 | 0.290986 |
| AMF x Population | 85.42 | 2 | 4.4881 | 0.017275 |
| Residuals | 390.16 | 41 |  |  |
| **Tissue N** |  |  |  |  |
| Intercept | 165.04 | 1 | 2.6251 | 0.112022 |
| AMF | 254.37 | 2 | 2.0230 | 0.143864 |
| Population | 231.86 | 1 | 3.6878 | 0.061023 |
| AMF x Population | 708.27 | 2 | 5.6328 | 0.006485 |
| Residuals | 2892.04 | 46 |  |  |


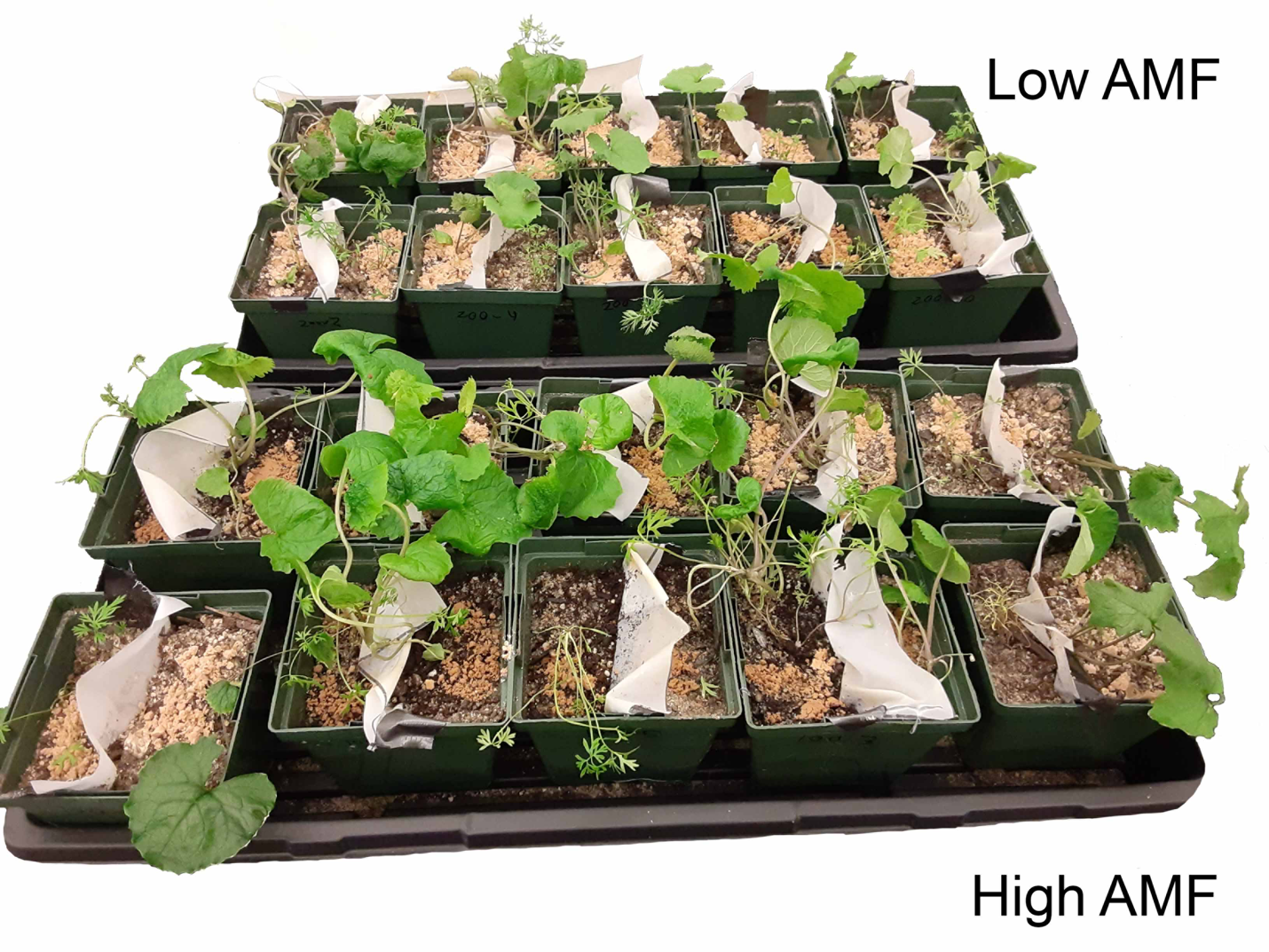


**Figure S1**. Photo of plants from the Drumlin farm population comparing effects of low versus high AMF additions on garlic mustard biomass. Garlic mustard plants grown in the high AMF inoculation treatment shown in the front have larger leaves and greater biomass compared to plants grown under conditions of low AMF inoculation shown in the back. This demonstrates the positive effects of AMF on garlic mustard growth.





**Figure S2**. Levels 4-methoxyindol-3-ylmethyl glucosinolate. (4MOI3M) and indol-3-ylmethyl glucosinolate (I3M) across the AMF inoculation treatments and between the two populations. The x-axis shows three different levels of AMF inoculation separated between the two populations from Black Rock and Drumlin Farm.


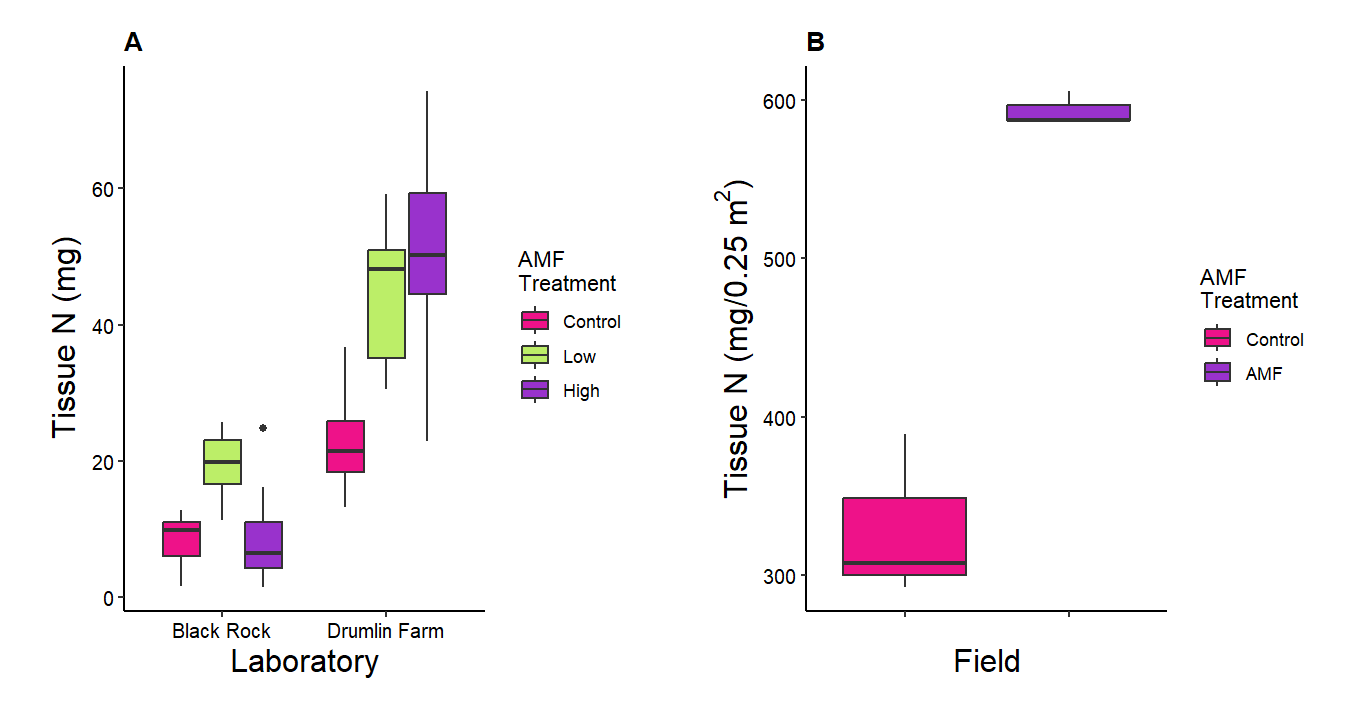


**Figure S3.** The effect of AMF inoculation on total tissue N in garlic. Tissue N increased with AMF inoculation in both the Black Rock (F(2)=9.58, p=0.001) and Drumlin Farm (F(2)=11, p<0.001) populations in the laboratory experiment (A) and in the field (B, H(1)=3.86, p=0.05).

**
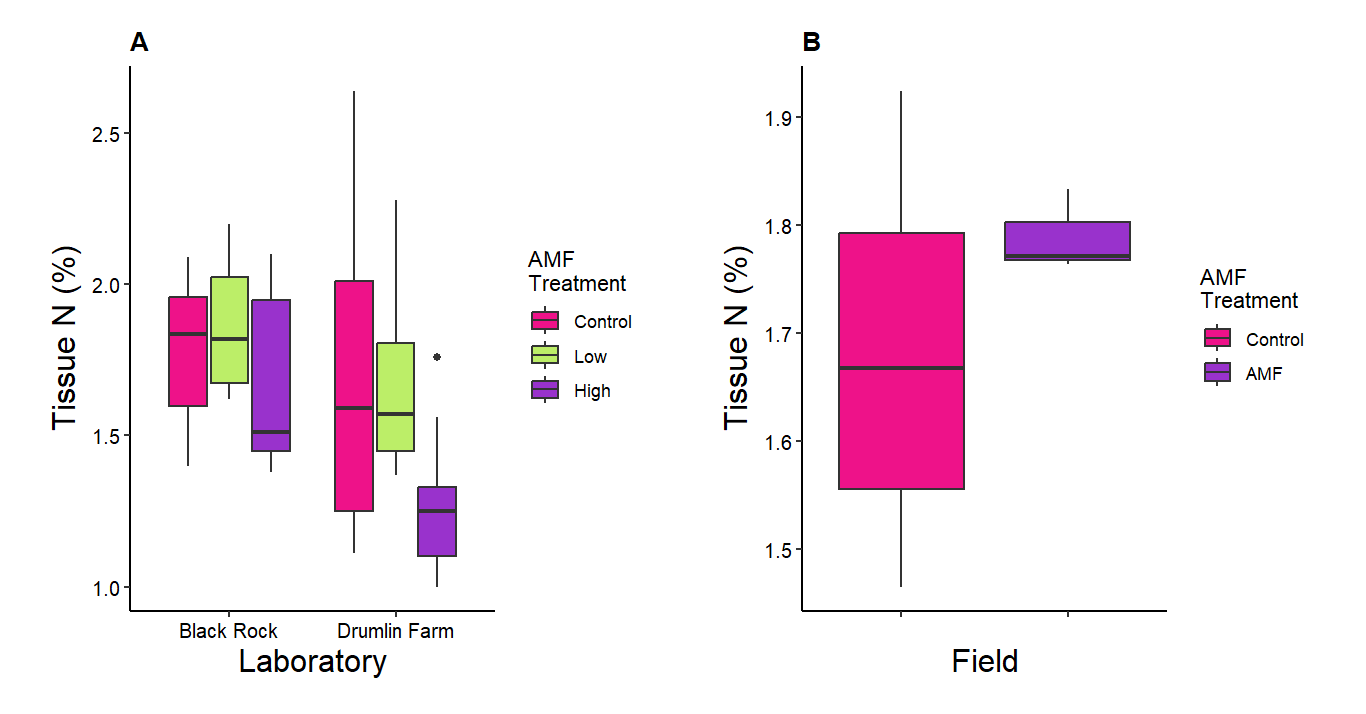
**

**Figure S4.** The effect of AMF inoculation on relative tissue N in garlic mustard. Relative tissue N did not differ significantly among treatments in either the laboratory (A) or field (B) experiments.


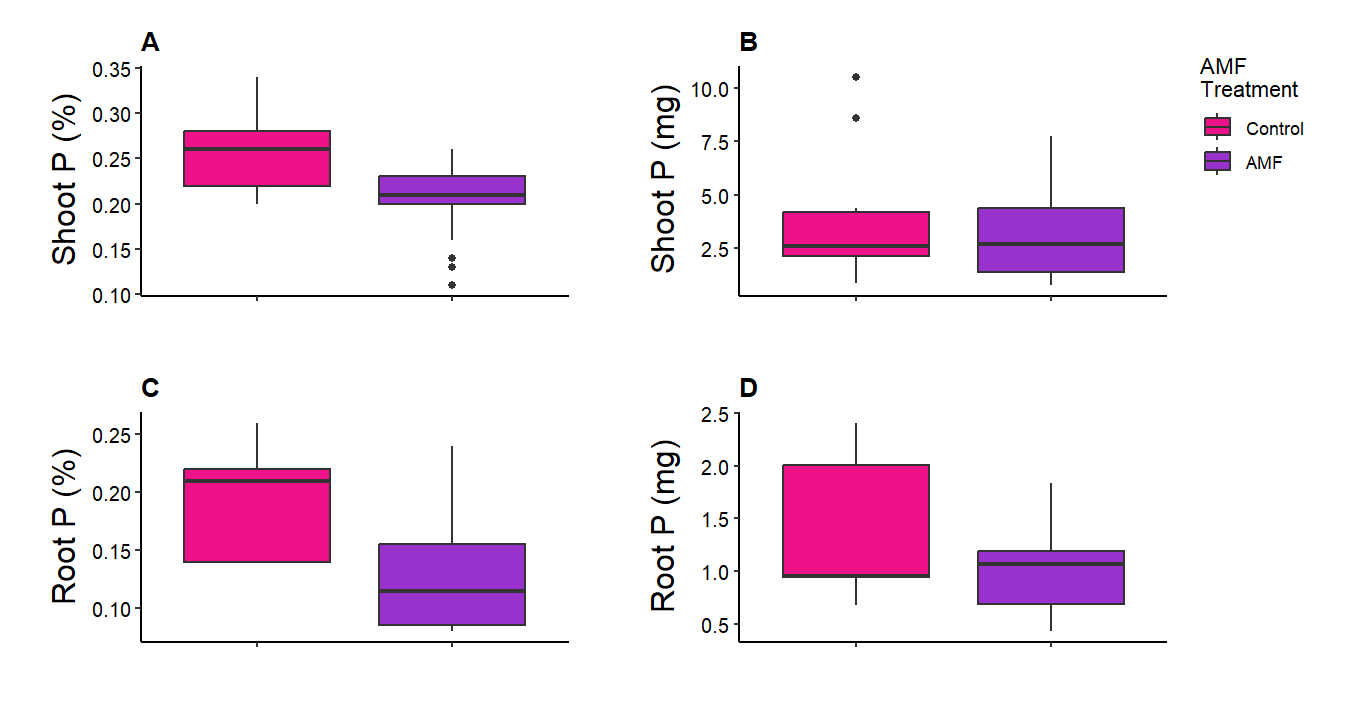


**Figure S5.** The effect of AMF inoculation in the field study on relative and absolute tissue P in garlic mustard *in situ*. Relative P was significantly lower in both shoot (A, H(1)= 9.986, p=0.0016) and root (C, H(1)=3.9, p=0.048) tissues in AMF treated plants. Absolute tissue P (B, D) did not differ between treatments.





**Figure S6.** There is no relationship between the adjacent host plant root arbuscule colonization and garlic mustard biomass (A) or root sinigrin production (B).
